# Employment status and work-related difficulties in stomach cancer survivors compared with the general population

**DOI:** 10.1038/sj.bjc.6604236

**Published:** 2008-02-19

**Authors:** M K Lee, K M Lee, J-M Bae, S Kim, Y-W Kim, K W Ryu, J H Lee, J-H Noh, T-S Sohn, S-K Hong, Y H Yun

**Affiliations:** 1 Research Institute and Hospital, National Cancer Center, 809 Madu dong, Ilsan gu, Goyang si, Gyeonggi do 411-769, Korea; 2 Department of Surgery, Samsung Medical Center, Sungkyunkwan University, School of Medicine, Seoul, Korea

**Keywords:** employment status, work, stomach cancer survivor, general population

## Abstract

Little was known about work situation and work-related difficulties, including housework after stomach cancer diagnosis. We aimed to compare employment status and work-related difficulties between stomach cancer survivors and the general population. We enrolled 408 stomach cancer survivors from two hospitals 28 months after diagnosis and 994 representative volunteers from the general population from 15 geographic districts. Working was defined as being employed (including self-employed) and nonworking as being retired or a homemaker. Nonworking was significantly higher among stomach cancer survivors (46.6%) than in the general population (36.5%). Compared with the general population, the survivors had more fatigue in performing both housework (adjusted odds ratio (aOR)=2.08; 95% confidence interval (95% CI)=1.01–4.29) and gainful work (aOR=4.02; 2.55–6.33). More cancer survivors had reduced working hours (aOR=1.42; 95% CI=4.60–28.35) and reduced work-related ability (aOR=6.11; 95% CI=3.64–10.27) than did the general population. The association of nonworking with older age and being female was significantly more positive for survivors than for the general population. Among survivors, poorer Eastern Cooperation Oncology Group Performance Status and receiving total gastrectomy were positively associated with nonworking. Stomach cancer survivors experienced more difficulties in both housework and gainful employment than did the general population. Our findings on stomach cancer survivors’ work-related difficulties and the predictors of nonworking will help physicians guide patients towards more realistic postsurgical employment plans.

Stomach cancer is the fourth most common malignancy in the world, with an estimated 870 000 new cases reported each year (Stewart *et al*, 2003). Over the past 20 years, early detection and treatment improvements have led to an increased number of long-term stomach cancer survivors (Roukos, 1999).

Returning to work and performing housework after recovering from cancer is important to the survivors’ family and social roles as well as to their finances. Cancer survivors may return to the work they did before diagnosis, but they may experience new physical limitations as a result of the disease and its treatment (Hewitt *et al*, 2003). Many studies have assessed the employment status of cancer survivors (Maunsell *et al*, 2004; Yabroff *et al*, 2004; Bednarek and Bradley, 2005; Bradley *et al*, 2005; Drolet *et al*, 2005; Short *et al*, 2005; Bouknight *et al*, 2006), but only four of them included comparison groups (Maunsell *et al*, 2004; Yabroff *et al*, 2004; Bradley *et al*, 2005; Drolet *et al*, 2005), which are crucial to detecting cancer-specific effects. Furthermore, the majority of studies focused on breast (Maunsell *et al* 2004; Drolet *et al*, 2005; Bouknight *et al*, 2006) or heterogeneous types of cancer (Bradley and Bednarek, 2002a; Yabroff *et al*, 2004; Bednarek and Bradley, 2005; Short *et al*, 2005) and did not consider the survivors’ ability to do housework. Little is known about the employment status and work-related difficulties associated with stomach cancer. The initial complications of its treatment, such as eating restrictions, weight loss, fatigue, and anxiety (Vickery *et al*, 2001; Bae *et al*, 2006), may diminish the long-term health-related quality of life. The effect of stomach cancer treatment on long-term health-related quality of life can limit or destroy the ability to work. To investigate this issue, we compared stomach cancer survivors’ employment status, workplace-related difficulties, housework-related difficulties, and correlates of not working with those of the general population.

## MATERIALS AND METHODS

### Study population

#### Survivors

We identified the patients for this cross-sectional study through the stomach (ICD code, C16) surgery database at the National Cancer Center and the Seoul Samsung Medical Center in Korea. Like the patients, representative of all hospitals in Korea, the study sample from the two hospitals resided in 15 geographic districts spread across the country. We collected information on the stage, type of surgery, time since surgery, history of cancer therapy, extent of lymphadenectomy, and recurrence from the hospital cancer registries. Eligibility required a diagnosis of stage I–III stomach cancer during 2001 or 2002 and being physically and mentally fit to fill out the questionnaire. Patients were excluded if they had a prior history of another cancer, could not speak Korean, or were <18 years old. We invited eligible patients to participate in the study by telephone, and those who agreed to participate were sent the questionnaire with consent forms and a postage-paid return envelope. Subjects who did not return the questionnaire within a month received a reminder card and a phone call by a staff member who explained the purpose of the study and requested participation. Interested subjects were asked to sign the informed consent form and to complete and return the questionnaire. Subjects who did not want to participate were asked their reasons. We reviewed the patient-reported questionnaires to check for missing or incomplete information and to confirm consistency between the clinical database and the self-reported data. When we found inconsistencies, we resolved them by telephonic communication with the family. We reflected the final confirmation as survivors’ data. Hospital records yielded 887 patients who had undergone curative surgery for stomach cancer during 2001 or 2002. Of those, 32 who had a prior history of another cancer or could not speak Korean, or who were <18 years old were excluded. Of the 855 potentially eligible remaining patients, 83 had died and 81 were not contacted. We were able to contact 691 patients by telephone. Of those, 97 refused to participate and 165 did not return the questionnaire. The most frequent reasons survivors gave for refusing to participate or to return the questionnaire were as follows (i) they thought completing the questionnaire was too time-consuming, (ii) they had no help in writing, or (iii) they regarded it as inconvenient or a violation of privacy.

Of the remaining 429 patients, 18 were excluded because they did not complete the questionnaire and three were excluded because they experienced a recurrence of cancer. That left 408 patients in the final sample (response rate, 59.0%).

#### General population

In each district, the survey was conducted in age and sex strata according to the guidelines of the 2000 Korean census. We selected villages and streets using the probability proportional to size (PPS) technique, which is widely used and is the recommended method for obtaining a representative national sample (Levy and Lemeshow, 1991). Probability proportional to size technique considers the size of individual groups and corrects for differences in the probability of larger and smaller groups being sampled. Eligibility criteria included being physically and mentally fit to fill out the questionnaire or communicate with the interviewer and being ⩾18 years old. The representative sample ⩾18 years old consisted of 2447 persons. The interviewers visited each person at home or in the workplace, evaluated the eligibility, and explained the purpose of the study to the eligible person. Of 2447 potentially eligible persons, 1447 refused to participate or did not complete the survey. The most frequent reasons people gave for refusing to participate were that they felt too busy to complete the questionnaire (*n*=734), that the survey was inconvenient (*n*=356), that they did not want to provide personal information (*n*=156), or others. One thousand of the eligible persons who agreed to participate completed the self-reported questionnaire in the presence of an interviewer who was there to explain the purpose of the study, but like the survivors, they completed the questionnaire for themselves without the interviewer's assistance. Of those who completed the survey, six had a history of cancer and were excluded. We enrolled 994 members of the eligible general population for the comparison group. All participants provided written informed consent. Although the response rate in this study (41%) was low, the sample appeared to be representative of the general population because the distribution of age and sex was similar to that of the 2000 Korean census (Yun *et al*, 2007).

### Study variables

We used a questionnaire to collect information on the employment status and socio-demographic characteristics of stomach cancer survivors and the general population. ‘Working’ was defined as being employed or self-employed and ‘nonworking’ as being retired or a homemaker. If participants were currently working, they were asked what kind of work-related difficulties they were having. The question included the following five multiple-choice items: (1) reduced working hours, (2) lessened work-related ability than before cancer diagnosis, (3) easily fatigued and exhausted, (4) reduced opportunity for promotion, and (5) decreased wages. If the items were not applicable, participants were asked to write in their work-related difficulties. If they were homemakers, they were asked what kind of housework-related difficulties they were having. The question included the following three multiple-choice items: (1) physically limited, (2) easily fatigued and exhausted but not physically limited, (3) emotionally distressed (such as feeling depressed or anxious). If the choice was not applicable, participants were asked to write in their housework-related difficulties. We did not specifically measure physical limitations as an item for work-related difficulties but used ‘reduced working hours’ and ‘lessened work-related ability than before’ as measures of work-related difficulties following physical limitations. If they were currently not working (except for homemakers), they were asked why they were not working. The question included the same three multiple-choice items used for homemakers (above). If the three multiple-choice items were not applicable, participants were asked to write in the reason for not working. We based most of the work-related questions on earlier studies (Maunsell *et al*, 2004; Yabroff *et al*, 2004; Bradley *et al*, 2005; Drolet *et al*, 2005; Short *et al*, 2005). Feasibility and comprehensibility of the full survey instrument – including work- and housework-related difficulties, reasons for unemployment, and socio-demographic and clinical characteristics – were pretested with 15 stomach cancer survivors in an outpatient clinic of the Korean National Cancer Center. Pretesting did not change the survey instrument, but no independent validation study was done.

### Statistical analysis

Differences of observed characteristics in the cancer survivors and general population can lead to biased estimates of the effect of cancer on employment. Therefore, we used propensity scores to balance the observable characteristics of the treatment (in our case, stomach cancer) and minimise bias in the selection of cases *vs* referent (Rosenbaum and Rubin, 1983). For an individual, propensity score is the probability of being treated (or, in this case, having stomach cancer) on the basis of observed characteristics (age, sex, education, marital status, religion, health cost financing, monthly household income, number of comorbidities, and number of family members), but score adjustment did not correct for differences between survivors and controls in unobserved characteristics. The propensity score is the estimated logistic regression model. Missing income data (*n*=74) were estimated by simple imputation via multivariate regression imputation. All estimates were robust as to whether we imputed the missing income data or excluded the imputed income data from the analysis. All statistical tests were two-sided. We used *t*-tests for continuous variables and *χ*^2^ tests for categorical variables in univariate analyses. We included variables with *P<*0.05 in univariate analyses in the multivariate logistic regression model, with a stepwise selection method.

### Ethics

The Institutional Review Boards of National Cancer Center and Samsung Medical Center approved the study.

## RESULTS

### Patient characteristics

The proportion of homemakers among the survivors (22.3%) and in the general population sample (22.7%) before propensity score adjustment did not differ significantly. Stomach cancer survivors differed significantly from the general population in several socio-demographic characteristics at baseline, but not after adjustment for propensity score (Table 1).

### Employment status of cancer survivors and general population

Table 2 shows survivors’ employment status at the time of diagnosis and at 28 months (range, 21–36 months) after diagnosis compared with the general population's employment status. The proportion working at the time of diagnosis was similar for stomach cancer survivors (65.9%) and the general population (63.5%). After diagnosis and treatment, however, the percentage of nonworking among survivors (46.6%) was higher than that among the general population (36.5%) (adjusted odds ratio (aOR)=1.75, 95% confidence interval (CI)=1.28–2.53). In the stratified analyses by age and sex, the proportion of cancer survivors working was currently significantly lower than at the time of diagnosis (respectively, male, 65.9 *vs* 85.8%; female, 18.3 *vs* 34.8%; <50-year-old, 64.6 *vs* 77.6%; 50- to 64-year-old, 57.6 *vs* 73.4%; ⩾65-year-old, 25.0 *vs* 53.1%. *P*<0.05 for all).

Physical limitation as the reason for nonworking was significantly higher for survivors than for the general population (aOR=7.68; 95% CI=3.64–10.27).

### Work- and housework-related difficulties

Table 3 shows work- and housework-related difficulties for cancer survivors and the general population. Compared with the general population, cancer survivors had a greater risk of reduced working hours (aOR=11.42; 95% CI=4.60–28.35) and work-related disability (aOR=6.11; 95% CI=3.64–10.27), and they were more easily fatigued and exhausted in the workplace (aOR=4.02; 95% CI=2.55–6.33). Those doing housework had more emotional distress (aOR=5.69; 95% CI=1.65–19.55) and were also more easily fatigued and exhausted (aOR=2.08; 95% CI=1.01–4.29).

### Associated factors with employment status in the survivors and general population

Table 4 shows the results of univariate analyses. In cancer survivors, age, sex, educational level, marital status, monthly household income, number of comorbidities, and number of family members were associated with employment status. In the general population, age, sex, educational level, marital status, having a religion, monthly household income, and number of comorbidities were associated with employment status.

Table 5 shows the results of multivariate logistic regression. In cancer survivors, being older (aOR=14.17, 95% CI=5.25–38.23) or female (aOR=16.83, 95% CI=8.30–34.11), having had a total gastrectomy (aOR=2.44, 95% CI=1.26–4.17), and having a poor ECOG performance status (aOR=2.12, 95% CI=1.19–3.78) were related to an increased probability of not being employed. In the general population, being older (aOR=6.02, 95% CI=3.26–10.99), female (aOR=5.43, 95% CI=3.99–7.32), or unmarried (aOR=2.00, 95% CI=1.44–2.79) and having two or more comorbidities (aOR=2.91, 95% CI=1.42–6.09) were related to an increased probability of not being employed. When we performed these analyses without adjusting for propensity score, the results were similar but the statistical power was less (data not shown).

## DISCUSSION

Most previous studies of cancer survivors’ employment status focused on breast or prostate cancer, which are common in Western Europe. To our knowledge, this is the first study to examine work- and housework-related difficulties and the correlates of employment status for stomach cancer survivors *vs* the general population. Although comparisons with previous studies may not be appropriate because of differences in length of follow-up, health care access, and disability laws, our finding that nonworking was 10% higher in stomach cancer survivors was similar to the findings of studies of breast cancer survivors and prostate cancer (Bradley *et al*, 2002b; Bradley *et al*, 2005; Drolet *et al*, 2005).

Our findings that stomach cancer survivors had difficulties in performing work due to increased fatigue and reduced work-capacity were also in agreement with findings from other studies (Stewart *et al*, 2001; Bradley and Bednarek, 2002a, Short *et al*, 2005). However, our findings were specific for stomach cancer survivors because we focused on comparing cancer survivors with the general population. Fatigue was a common problem in performing both housework and gainful work. Thus, even homemakers who did not work competitively or have assigned responsibilities experienced more fatigue than their counterparts in the general population. Because survivors may not be able to perform their normal home chores (Collins *et al*, 2004), their family role could change. This has been studied before among women with cancer (Collins *et al*, 2004; Serin *et al*, 2005). concluded that fatigue and anxiety were the most frequent problems for breast and gynaecological cancer patients, and those problems made housework more difficult (Zakowski *et al*, 2003). There were no reports of emotional distress being a work-related difficulty among workers and only two reports of it being the reason for current unemployment among nonworkers. Homemakers diagnosed with stomach cancer, however, might be more depressed than homemakers in the general population. We discuss the finding that having cancer and being a homemaker and balancing these two roles may be more difficult for stomach cancer survivors than for the general population. Families with a cancer patient may need to provide an emotional support system for them.

Our finding that older age and being female were common correlates of postcancer work cessation also agreed with previous results (Lash and Silliman, 2000; Spelten *et al*, 2003; Drolet *et al*, 2005), but we showed that this was the case relative to the general population. The correlation with older age may reflect the fact that the cancer occurred at a time of life when patients may have already been thinking about retirement or working less (Lash and Silliman, 2000; Drolet *et al*, 2005) and that greater physical limitations increased the tendency to stop working.

Our observation that women were less likely than men to work after cancer is in keeping with a previous study showing that women had more cancer-related disabilities than men (Short *et al*, 2005). Female stomach cancer survivors might find work difficult and attribute their work problems, or their personal decision to work less, to their disease (Yabroff *et al*, 2004). Our observation that 24% of male survivors but 48% of female survivors decreased their working hours may reflect that women value work less than men, perhaps because of family commitments or of not being the main earner, as discussed in a growing body of literature concerning changes in the values of cancer survivors (Yabroff *et al*, 2004).

Our finding that nonworking was associated with a number of comorbidities in the general population but not in cancer survivors has been reported in two previous studies (Bradley *et al*, 2002b). For cancer survivors, deciding whether to work is associated with their cancer rather than their other morbidities (Bradley *et al*, 2002b).

Our finding that nonworking was significantly greater among those who received total gastrectomy than among those who received subtotal gastrectomy may be due to the side effects of the surgery, such as eating restrictions and weight loss, which may have negative effects on getting along in the workplace (Vickery *et al*, 2001; Bae *et al*, 2006). The fact that the type of surgery can affect a patient's ability to work after recovery should be considered in treatment decisions.

We found that physical limitation is the main correlate of not working and it may be caused by the fact that ECOG performance status was highly correlated with physical functioning (Kobayashi *et al*, 1998).

This study had several limitations. First, it may have been subject to selection bias, but to the extent that we could verify it, there seemed to be no systematic differences between participants and those we intended to recruit. Because the study sample was drawn from two hospitals while the control sample was drawn from 15 geographic districts, the health care market might be different for each group. However, the study sample from the two hospitals was distributed all across the country. In a complementary analysis, the distribution of districts in both samples was not different (*P*=0.22, data not shown). Additionally, we corrected for the different distribution of socio-demographic characteristics between the two groups by propensity score adjustment. That allowed for better control than was evident in studies that matched groups for only a few characteristics, such as age and education. Second, we used different recruiting methods for the two groups, but the eligibility requirements and self-reported questionnaire were the same for both. Third, the response rate of the general population (41%) and the survivors (59%) was relatively low. Because the reasons for refusal in both groups were unrelated to health problems or employment status, the fact that respondents differed from nonrespondents in having a greater male-to-female ratio, younger age, and a greater proportion of patients with early-stage disease was not likely to affect our results. Moreover, the low response rate was not likely to have influenced the findings in terms of working status because the employment rate of both the cancer survivors (66.2%) and the general population sample (63.5%) was similar to the employment rate of the Korean population (63%) during the time of the study (Korea Statistical Information System, 2005).

We found that stomach cancer survivors had difficulties at work: due to increased fatigue and reduced capacity, and that the type of surgery received appeared to play a role. We believe that our findings will help stomach cancer patients make more realistic postsurgical employment plans. In addition, the information can inform occupational rehabilitation programmes, occupational health services, and employers and guide government policy for stomach cancer survivors.

## Figures and Tables

**Table 1:** Sociodemographic characteristics of stomach cancer survivors and general population before and after propensity score adjustment

	**Cancer survivors (*N*=408) *n* (%)**	**General population (*N*=994) *n* (%)**	**Wald F^a^ (*P*-value)**	**Wald F^a^ adjusted for propensity score^b^ (*P*-value)**
*Age*				
⩽49	140 (34.3)	711 (71.5)		
50–64	194 (47.5)	206 (20.7)		
⩾65	74 (18.2)	77 (7.9)	167.9 (<0.001)	0.0001 (0.99)
				
*Sex*				
Male	300 (73.5)	497 (50.0)		
Female	108 (26.5)	497 (50.0)	65.2 (<0.001)	0.23 (0.62)
				
*Education*				
Less than high school graduate	171 (42.6)	161 (16.2)		
High school graduate or more	230 (57.4)	833 (83.8)	110.1 (<0.001)	0.07 (0.77)
				
*Marital status*				
Married	352 (88.6)	706 (71.0)		
Widowed/divorced/separated/single	45 (11.4)	288 (29.0)	48.4 (<0.001)	1.88 (0.16)
				
*Place of residence*				
Metropolitan	218 (46.4)	426 (42.9)		
City/country	269 (53.6)	568 (57.1)	1.5 (0.22)	0.7 (0.40)
				
*Having a religion*				
Yes	281 (69.9)	532 (53.5)		
No	121 (30.1)	462 (46.5)	31.5 (<0.001)	0.23 (0.62)
				
*Health cost financing*				
Health insurance	287 (71.9)	959 (96.6)		
Medical aid	112 (28.1)	34 (3.4)	184.1 (<0.001)	0.16 (0.68)
				
*Monthly household income, $US*				
<2000	143 (36.5)	243 (24.5)		
⩾2000	249 (63.5)	750 (75.5)	20.1 (<0.001)	0.11 (0.73)
				
*No. of comorbidities*				
0	215 (52.7)	738 (74.3)		
1	157 (38.5)	206 (20.7)		
⩾2	38 (8.8)	50 (5.0)	61.7 (<0.001)	0.003 (0.95)
				
*No. of family members*				
⩽3	284 (70.3)	751 (75.6)		
⩾4	116 (29.7)	243 (24.4)	4.0 (0.04)	0.08 (0.76)
				
*Current employment*				
Self-employed	140 (34.3)	264 (26.6)		
Employed	78 (19.1)	367 (36.9)		
Full-time worker	60 (76.9)	301 (82.0)		
Unemployed/retired	99 (24.3)	137 (13.8)		
Homemaker	91 (22.3)	226 (22.7)	37.4 (<0.001)	0.68 (0.38)
				
*Employment at the time of diagnosis*				
Self-employed	146 (35.8)	—		
Employed	123 (30.2)	—		
Full-time worker	58 (47.2)			
Unemployed /retired	66 (16.1)	—		
Homemaker	73 (17.9)	—	N/A	N/A

Abbreviations: N/A=not applicable.

a F statistics based on Wald *χ^2^*.

b The propensity score summarizes the differences in observable characteristics between cancer survivors and general population, that is, age, sex, education, marital status, religion, monthly household income, health cost financing, number of family members, and number of comorbidities.

**Table 2:** Model-based adjusted odds ratio for not working for stomach cancer survivors (1) compared with the general population and (2) currently compared with the time of diagnosis

	**Working *n* (%)**	**Not working *n* (%)**	**aOR (95% CI)^a^**	**aOR (95% CI)^b^**	**aOR (95% CI)^c^**
*General population (n=994)*					
Current employment status	631 (63.5)	363 (36.5)	1 (referent)	—	1 (referent)
					
*Cancer survivors (n=408)*					
Employment status at diagnosis	269 (65.9)	139 (34.1)	0.75 (0.53-1.08)	1 (referent)	—
Current employment status	218 (53.4)	190 (46.6)	—	2.26 (1.61–3.15)	1.75 (1.28–2.53)

Abbreviations: aOR=adjusted odds ratio.

a aOR for not working at the time of diagnosis for cancer survivors compared with current employment status in the general population, adjusted for age, sex, education, marital status, religion, monthly household income, type of health cost financing, number of family members, number of comorbidities, and propensity score.

b aOR for cancer survivors of currently not working compared with their employment status at the time of diagnosis, adjusted for age.

c aOR for currently not working for cancer survivors *vs* the general population, adjusted for age, sex, education, marital status, religion, monthly household income, type of health cost financing, number of family members, number of comorbidities, and propensity score.

**Table 3:** The comparison^a^ of work-related difficulties between the stomach cancer survivors and the general population

	**General population (*N*=994)**	**Cancer survivors (*N*=408)**
Work-related difficulties experienced by worker	*n*=631	*n*=218
Reduced working hours, *n* (%)	13 (2.1)	22 (13.6)
aOR (95% CI)	1 (referent)	11.42 (4.60–28.35)
Lessened work-related ability than before^b^ *n* (%)	67 (10.6)	60 (37.0)
aOR (95% CI)	1 (referent)	6.11 (3.64–10.27)
Easily fatigued and exhausted, *n* (%)	141 (22.4)	81 (50.0)
aOR (95% CI)	1 (referent)	4.02 (2.55–6.33)
Reduced opportunity for promotion, *n* (%)	66 (10.5)	7 (4.3)
aOR (95% CI)	1 (referent)	0.45 (0.17–1.17)
Decreased wages, *n* (%)	253 (40.1)	50 (30.9)
aOR (95% CI)	1 (referent)	0.71 (0.45–1.10)
		
Housework-related difficulties experienced by home maker	*n*=226	*n*=91
Emotional distress (depression or anxiety), *n* (%)	9 (4.0)	10 (12.7)
aOR (95% CI)	1 (referent)	5.69 (1.65–19.55)
Easily fatigued and exhausted but no physical limitation, *n* (%)	131 (58.0)	58 (73.4)
aOR (95% CI)	1 (referent)	2.08 (1.01–4.29)
Physical limitations, *n* (%)	38 (16.8)	11 (13.9)
aOR (95% CI)	1 (referent)	0.86 (0.30–2.45)
		
Reasons for non-working	*n*=363	*n*=190
Physical limitations, *n* (%)	7 (1.9)	40 (21.1)
aOR (95% CI)	1 (referent)	7.68 (3.64–10.27)
Easily fatigued and exhausted but no physical limitation, *n* (%)	16 (4.4)	24 (12.6)
aOR (95% CI)	1 (referent)	1.84 (0.70–4.88)
Emotional distress (depression or anxiety), *n* (%)	0 (0.0)	2 (1.1)
aOR (95% CI)	1 (referent)	N/A
Etc^c^, *n* (%)	28 (7.7)	29 (15.3)
aOR (95% CI)	1 (referent)	1.75 (0.34–3.68)

Abbreviations: aOR=adjusted odds ratio; CI=confidence interval; N/A=Not Available.

a aOR for general population *vs* cancer survivors, adjusted for age, sex, education, marital status, religion, monthly household income, type of health cost financing, number of family members, number of comorbidities, and propensity score.

b The item was ‘Lessened work-related ability than before cancer diagnosis’ for survivors and ‘Lessened work-related ability than before; for the general population.

c Etc includes ‘not wanting to work’ and ‘not having been employed since the previous time’.

**Table 4:** The univariate results of relationship between employment status in stomach cancer survivors and general population

	**Cancer survivors**			**General population**		
**Characteristic**	**Working *n* (%)**	**Not working *n* (%)**	***P***	**Working *n* (%)**	**Not working *n* (%)**	***P***
**Sociodemographic factors**						
*Age (year)*						
Mean (SD)	51.4 (8.7)	57.6 (11.4)	<0.001	40.7 (11.5)	43.9 (17.3)	0.002
⩽49	92 (65.7)	48 (34.3)		487 (68.5)	224 (31.5)	
50–64	112 (57.3)	82 (42.3)		124 (60.2)	82 (39.8)	
⩾65	14 (18.9)	60 (81.1)	<0.001	20 (26.0)	57 (74.0)	<0.001
						
*Sex*						
Male	200 (66.7)	100 (33.3)		402 (80.9)	95 (19.1)	
Female	18 (16.7)	90 (83.3)	<0.001	229 (46.1)	268 (53.9)	<0.001
						
*Education*						
Less than high school graduate	69 (40.4)	102 (59.6)		64 (39.8)	97 (60.3)	
High school graduate or more	145 (63.0)	85 (40.0)	<0.001	567 (68.1)	266 (31.9)	<0.001
						
*Marital status*						
With spouse	199 (56.5)	153 (43.5)		478 (67.7)	228 (32.3)	
No spouse	15 (33.3)	30 (66.7)	0.003	153 (53.1)	135 (46.9)	<0.001
						
*Place of residence*						
Metropolitan area	65 (49.2)	67 (50.8)		310 (64.3)	172 (35.7)	
City/country	151 (56.1)	118 (43.9)	0.19	321 (62.7)	191 (37.3)	0.59
						
*Having a religion*						
Yes	153 (54.5)	128 (45.6)		315 (59.2)	217 (40.8)	
No	62 (51.2)	59 (49.8)	0.55	316 (68.4)	146 (31.6)	0.002
						
*Monthly household income, $US*						
<2000	54 (3783)	89 (62.2)		141 (58.0)	102 (42.0)	
⩾2000	160 (64.3)	89 (35.7)	<0.001	488 (65.6)	125 (34.4)	0.03
						
*No. of comorbidities*						
0	117 (54.4)	98 (45.6)		499 (67.6)	239 (32.4)	
1	89 (56.7)	68 (43.3)		117 (56.8)	89 (43.2)	
⩾2	12 (33.3)	24 (66.7)	0.03	15 (30.0)	35 (70.0)	<0.001
						
*No. of family members ⩾18 yr*						
⩽3	136 (49.6)	138 (50.4)		479 (63.8)	272 (36.2)	
⩾4	76 (65.5)	40 (34.5)	0.004	152 (62.5)	91 (37.5)	0.72
						
**Clinical factor**						
*Time since operation*						
Mean (SD), months	27.9 (3.5)	28 (3.6)	0.95	N/A	—	—
						
*Type of surgery*						
Subtotal gastrectomy	183 (57.5)	135 (42.5)				
Total gastrectomy	33 (38.0)	54 (62.0)	0.001	N/A	—	—
						
*Stage*						
I or II	197 (54.4)	165 (45.6)				
III	19 (46.3)	22 (53.7)	0.32	N/A	—	—
						
*Dissection*						
Limited lymphadenectomy	6 (60.0)	4 (40.0)				
Extended lymphadenectomy	210 (53.8)	180 (46.2)	0.96	N/A	—	—
						
*Received radiation*						
Yes	26 (54.2)	22 (45.8)				
No	181 (52.9)	161 (47.1)	0.87	N/A	—	—
						
*Received chemotherapy*						
Yes	49 (45.4)	59 (54.6)				
No	157 (55.3)	127 (44.7)	0.07	N/A	—	—
						
*ECOG PS* a						
1	156 (63.7)	89 (36.3)				
2∼4	53 (37.1)	90 (62.9)	<0.001	N/A	—	—
						
*Time since operation, months*						
<24	192 (53.5)	167 (46.5)				
⩾24	26 (53.1)	23 (46.9)	0.95	N/A	—	—

Abbreviations: ECOG PS=Eastern Cooperation Oncology Group Performance Status; N/A=not applicable.

a ECOG PS grades: 1, Restricted in physically strenuous activity but ambulatory and able to carry out work of a light or sedentary nature, for example, light house work, office work; 2, Ambulatory and capable of all self care but unable to carry out any work activities. Up and about more than 50% of waking hours; 3, Capable of only limited selfcare, confined to bed or chair more than 50% of waking hours; 4, Completely disabled. Cannot carry on any self care. Totally confined to bed or chair.

**Table 5:** Model-based adjusted odds ratio^a^ of not working by logistic regression analysis with the stepwise method in cancer survivors and general population

	**Cancer survivors (*N*=408)**		**General population (*N*=994**)	
**Characteristic**	**aOR for not working (95% CI)**	***P***	**aOR for not working (95% CI)**	***P***
*Age*				
⩽49	1 (referent)		1 (referent)	
50–64	2.51 (1.28–4.91)	0.007	1.50 (1.02–2.14)	0.04
⩾65	14.17 (5.25–38.23)	<0.001	6.02 (3.26–10.99)	<0.001
*Sex*				
Male	1 (referent)		1 (referent)	
Female	16.83 (8.30–34.11)	<0.001	5.43 (3.99–7.32)	<0.001
*Marital status*				
With spouse	1 (referent)		1 (referent)	
No spouse	1.88 (0.57–6.23)	0.230	2.00 (1.44–2.79)	<0.001
				
*No. of comorbidities*				
0	1 (referent)		1 (referent)	
1	0.64 (0.21–1.96)	0.328	0.83 (0.40–1.74)	0.617
⩾2	1.20 (0.63–2.27)	0.275	2.91 (1.42–6.09)	0.004
				
*Type of surgery*				
Subtotal gastrectomy	1 (referent)			
Total gastrectomy	2.44 (1.26–4.17)	0.007	N/A	
				
*ECOG PS*				
1	1 (referent)			
2∼4	2.12 (1.19–3.78)	0.01	N/A	—

Abbreviations: (aOR)=Model-based adjusted odds ratios, ECOG PS=Eastern Cooperation Oncology Group Performance Status; N/A=not applicable.

a Model-based adjusted odds ratios (aOR) are from a series of logistic regression models with stepwise method whose covariates were statistically significant (*P*<0.05) in univariate analyses (Table 4).
